# Importance of male partner’s involvement in prevention of mother to child transmission of HIV/AIDS in Ethiopia: a systematic review and meta analysis until June 2021

**DOI:** 10.1186/s13690-022-00971-7

**Published:** 2022-10-19

**Authors:** Shimeles Biru Zewude, Asrat Hailu Dagne, Tewodros Magegnet Ajebe

**Affiliations:** 1 Department of Midwifery, College of Medicine and Health Sciences, Debre Tabor University, Debre Tabor, Ethiopia; 2 Department of Midwifery, College Of Medicine and Health Sciences, Wollo University, Dessie, Ethiopia

**Keywords:** Male partner, Prevention of mother-to-child transmission of HIV, Meta analysis, Ethiopia

## Abstract

**Background:**

To promote the prevention of mother-to-child transmission (PMTCT) of HIV/AIDS male partners play a significant role, especially in developing country were men’s are a decision maker in domestic activity. When men are involved in PMTCT it would boost the service utilization and uptake of ART drugs. Thus this systematic review and Meta analysis aims to assess importance of male partner involvement in prevention of mother to child transmission of HIV/AIDS in Ethiopia.

**Methods:**

Studies were accessed through an electronic web-based search mechanism from PubMed, Advanced Google Scholar, WHO databases and journals (African Health Monitor, Pan African Journal of Public Health), using independent and combinations of key terms together with a reference list of included studies. Two reviewers independently screened and assessed the quality of studies based on pre-specified criteria. When a disagreement between the two reviewers happened; the third reviewer was invited and resolve it based on the stated objectives and inclusion criteria. Measures of effects were pooled and random effect meta analysis was conducted.

**Results:**

Ten studies met the inclusion criteria. The pooled prevalence of male involvement in PMTCT was 31.8% (95% CI; 22.3–41.3 I^2^ = 98.4%, *p* = 0.00). being urban residence(AOR = 2.43 95%CI;1.42–4.18), partner knowledge (AOR = 2.84 95%CI;1.90–4.22), knowledge on Antenatal care(AOR = 3.5 95%CI;1.80–6.76), partners who had no negative perception towards for PMTCT (AOR = 3.21 95%CI;2.18–4.72), government employee(AOR = 2.57 95%CI;1.76–3.75), partners informed of need to go for PMTCT(AOR = 3.83 95%CI;1.88–7.79), health institution related barriers(AOR = 2.6 95%CI;1.882–3.622), primary (AOR = 2.21 95%CI;1.29–3.80), and secondary education(AOR = 2.67 95%CI;1.69–4.19) were significant factors related with male partner involvement in prevention of mother to child transmission of HIV/AIDS.

**Conclusion:**

The proportion of male involvement in the Prevention of mother-to-child transmission of HIV in Ethiopia was low. Interventions aimed at improving male participation in the Prevention of mother-to-child transmission should consider the factors related to it. Healthcare services may need to be inclusive and could help men active engagement in PMTCT programs.

**Supplementary Information:**

The online version contains supplementary material available at 10.1186/s13690-022-00971-7.

## Background

HIV/AIDS is one of the pandemic public health problems affecting many people including children. More than 38 million people globally were living with HIV in 2019 [1, 2]. Worldwide 120,000 children died due to AIDS-related illnesses in 2016. This equates to 328 deaths every day. This is despite a 62% reduction in AIDS related death since 20 years ago. In the same year, 24% of pregnant women living with HIV did not have access to ARVs to prevent transmission to their infants and 160,000 children become infected with HIV [3]. Form the overall 90% of new infection believed to stem from mother to child transmission (MTCT) during pregnancy, childbirth and/or post-partum [4]

Male involvement has been recognized as a priority focus area to be strengthened in PMTCT but testing male partners for HIV in the context of preventing mothers to child transmission remains a challenge in most low and middle-income countries[5]. Male involvement has a great role in preventing women’s risk of infection by HIV, utilization on her PMTCT, to revive medication and helping women’s for proper infant feeding practices [6, 7]. Another advantage of partner involvement in PMTCT is that there is an over-growing discordant rate among the couple. In Ethiopia, 5.6% of HIV negative urban dowelling married men are living with HIV infected partners and 2.2% of married HIV negative women are living with infected husbands, this implies that only screening the mother to prevent mother to child transmission of HIV doesn’t safeguard the child from acquiring HIV [8, 9].

Countries are plan to nullifying mother to child transmission of HIV by 2030 [10]. To achieve the aforementioned goal male involvement is highly demanded. Poor male participation could also contribute to the problem as women in developing counties may depend on partners' decisions and support for both HIV testing and adherence to recommended PMTCT interventions [11]. Sub-optimal male participation is considered as a bottleneck for PMTCT programmatic achievement and has a negative bearing on women’s participation in voluntary HIV counseling and testing (HCT) and further program enrolment. Joint counseling and HIV testing for couples seem to be key to success in this regard [12]. At the national level there is no clear evidence that could be attributable factors associated with male partner involvement in the uptake of PMTCT services. Therefore, this systematic review and meta-analysis was aimed to address the following review question;

What is the proportion of male involvement in prevention of mother to child transmission of HIV/AIDS?

What are the factors associated with male partner involvement in the prevention of mother to child transmission of HIV/AIDS?

## Methods

### Inclusion and exclusion criteria

The report was written by using preferred reporting items for systematic reviews and Meta analysis (PRISMA) guideline [13]. (additional file 1) we considered articles published in the English language, have relevant full-text form, and studied in Ethiopia. There was no restriction on the publication period. The outcome was the proportion of male involvement in PMTCT as a decision-making tool, and its predictive factors, both published and unpublished studies at any time were included. However, studies available only as abstract with unclear outcomes, commentaries, editorials, and reviews were excluded.

### Information sources and search strategies

Studies were accessed through the electronic web-based search mechanism from PubMed, Advanced Google Scholar, WHO databases and journals (African Health Monitor, Pan African Journal of Public Health). In addition, further search was made through snowballing or retrieving from relevant references used in related studies. A combination of medical subject headings (MeSH terms) using Boolean operators and keywords related to importance of Male partner involvement in PMTCT were used to search studies. We included articles published from inception until 10 June 2021. (Additional file 2).

### Study selection process

Retrieved studies were exported to reference manager software, endnote version 6. Duplicated studies were removed using the endnote and manually. Two independent reviewers (SB) and (AH) screened the title and abstract for relevance. During this preliminary assessment, primary studies found to be irrelevant were excluded. When a disagreement between two reviewers happened, the third reviewer (TM) was used to handle the disagreement based on the relevance of the stated objectives and inclusion criteria. Finally studies with relevant information and fulfill inclusion criteria were selected for full-text review and excluded study were reason out via flow chart.

### Data collection process

The data extraction form was prepared using an excel spreadsheet. Two independent reviewers extracted data by using a structured data extraction form. The name of the first author and year, region, study area, study design, sample size, determinants of male involvement, AOR (95% CI) were extracted. The extracted data then edited and saved in a comma-delimited file to suit for the analysis.

### Measurement of outcomes

It has two main outcomes; proportion of male involvement in prevention of mother to child transmission of HIV/AIDS and the second one is importance of male partner involvement in prevention of mother to child transmission of HIV/AIDS. The odds ratio was calculated for the common risk factors of the reported studies.

### Study risk of bias assessment

Two independent reviewers (SB) and (AH) appraised the quality of each study. The Joanna Briggs Institute (JBI) quality appraisal checklist was applied [14]. Data extraction was considered when studies scored above 50% for JBI quality indicators (Additional file 3). The inter-rater agreement between authors for study inclusion, data extraction, and methodological quality was assessed using Cohen’s kappa coefficient (values ≤ 0 as indicating no agreement and 0.01–0.20 as none to slight, 0.21–0.40 as fair, 0.41– 0.60 as moderate, 0.61–0.80 as substantial, and 0.81–1.00 as almost perfect agreement) [15]. Substantial agreement between reviewers, i.e., Cohen’s kappa coefficient > 0.72, was accepted.

### Data synthesis and analysis

The data extracted and saved in comma-delimited file format in an excel spreadsheet was imported to Stata version 14.0 (Stata Corporation, College Station, TX, USA) statistical software for analysis. Heterogeneity among studies was checked using visual inspection of the results and using the I-squared (I^2^) statistic, in which 25, 50 and 75% represented low, moderate, and high heterogeneity respectively [16]. In this study I^2^ value of less than 50% was considered to interpret the combined effect size. Since there is heterogeneity between studies so Random effect model was used. Pooled analysis was done using mantel haenszel (M-H) statistical methods and effect measure was computed by odds ratio. Subgroup analysis was applied to investigate possible causes of statistical heterogeneity. Subgroup analysis was done by using publication year and sample size. Sensitivity analyses have been conducted to see the effects of a single study on determinants of male involvement in PMTCT. Potential publication bias was checked by using funnel plot and egger's regression test.

## Results

### Selection of studies

**Fig. 1:**
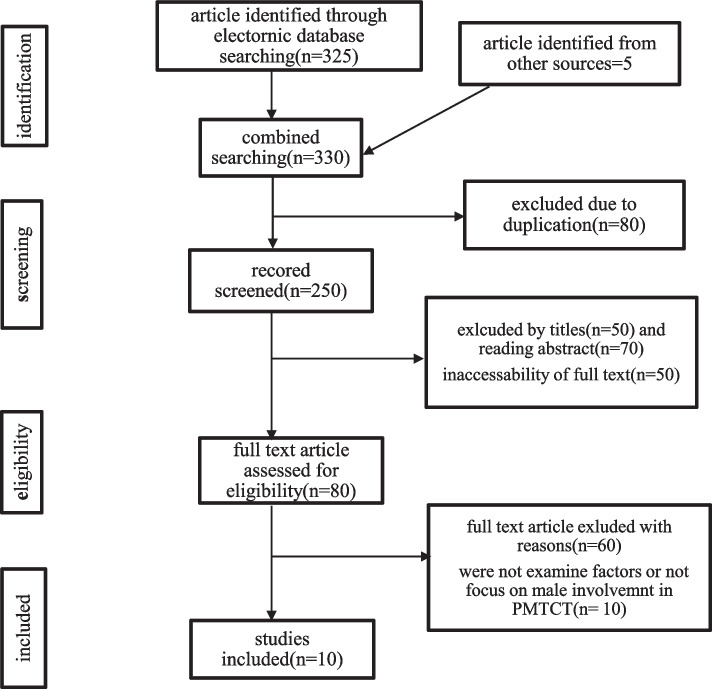
Flow chart showing search results of study selection procedure for the systematic review and Meta analysis of male involvement in prevention of mother to child transmission of HIV in Ethiopia until June 2021

A total of 330 studies were retrieved from sources by using pre-specified search strategies. All searched articles were exported to the endnote and then 80 were removed due to duplication. 250 Studies were screened for eligibility, relevance, accessibility, and outcome of interest. Accordingly, 220 studies were excluded due to inappropriate titles and were not examine factors or not focus on male involvement, and 15 is not original article and other reason. 5 articles were removed due to different outcomes of interest. Finally, 10 studies were included in the review. (Fig. 1).

### Study characteristics

Different factors such as urban residence, knowledge in PMTCT, partner knowledge of ANC, partners have no negative perception, government employee, partners which were informed of need to go for PMTCT, health institution related barriers, primary education, and secondary education were included in this study. A total of 4945 participants were included in this review. The sample size considered for primary studies ranges from 272 to 808. Regarding the study area, four of the studies were conducted at Amhara region, three in SNNPR (South Nation Nationalities and People region), and one in each region of Addis Ababa, Tigray, and Oromiya.

### Publication bias

**Fig. 2:**
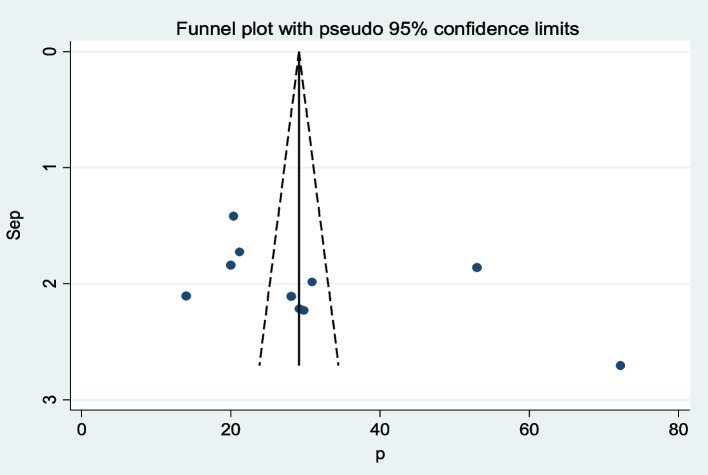
Funnel plot of prevalence of male involvement in Prevention of mother-to-child transmission of HIV in Ethiopia. Each dot represents a single study. The y-axis is the standard error of the effect estimate. The x-axis is transformed effect size. Asymmetrical distribution of dots on the both sides of the vertical line inside the triangle (funnel) shows relatively publication bias (subjective)

The funnel plot was assessed for asymmetry distribution of the magnitude of male involvement in PMTCT by visual inspection (Fig. 2) and Egger’s regression test showed (*P* = 0.12) indicate no evidence of publication bias.

### Pooled estimate of male involvement in PMTCT

**Fig. 3:**
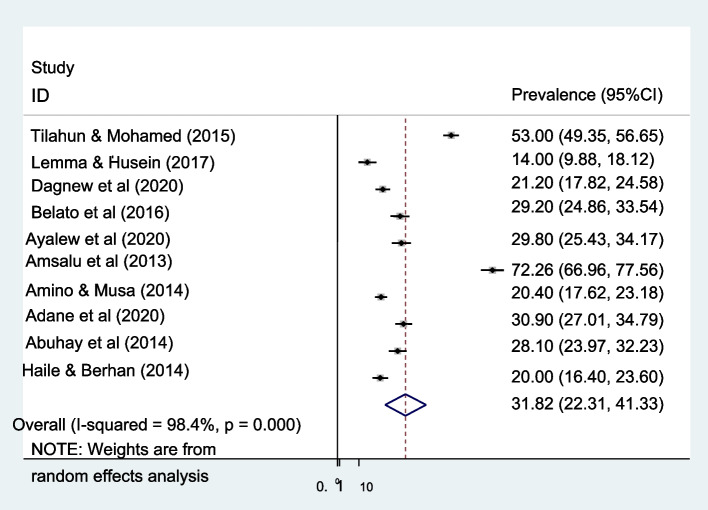
Forest plot showing heterogeneity among included studies to estimate male involvement in Prevention of mother-to-child transmission of HIV in Ethiopia. Each squared box indicates the sample size of individual studies. The horizontal line at the middle of each box indicates the 95% confidence interval of individual studies. The diamond shape at the end of the broken vertical line indicates the pooled effect size

The overall pooled prevalence of male partner involvement in PMTCT is presented with a forest plot (Fig. 3). Therefore, the estimated proportion of men’s involved in PMTCT in Ethiopia was 31.82% (95% CI 22.31–41.33 I^2^ = 98.4%, *P *< 0.000).

### Subgroup analysis

**Fig. 4:**
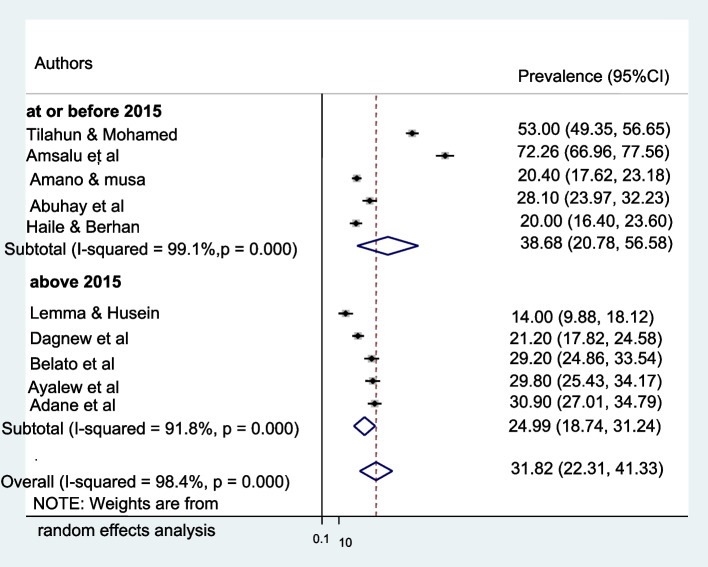
Subgroup analysis of the pooled prevalence of male involvement in Prevention of mother-to-child transmission of HIV based on the year of study in Ethiopia until June 2021

**Fig. 5:**
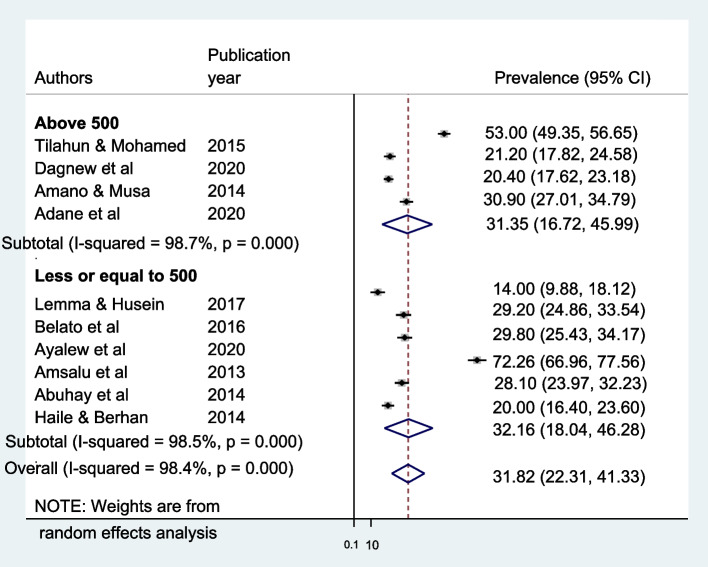
Subgroup analysis of the pooled prevalence of male involvement in Prevention of mother-to-child transmission of HIV based on the sample size in Ethiopia until June 2021

Subgroup analysis was employed with the evidence of heterogeneity. In this study, the Cochrane I^2^ statistic was 98.4%, *P* < 0.000, which showed evidence of marked heterogeneity. Therefore, subgroup analysis was done using the publication year and sample size. As a result, male involvement in PMTCT is highest in the study conducted at or before 2015, 38.68%, whereas 32.16% in sample size less than or equal to five hundred (Fig. 4 and 5).

### Sensitivity analysis

**Fig. 6:**
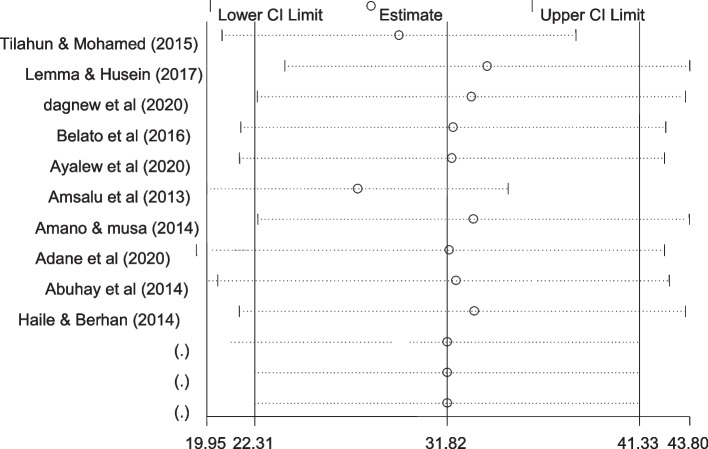
Sensitivity analysis of the pooled prevalence of male involvement in Prevention of mother-to-child transmission of HIV in Ethiopia until June 2021. Sensitivity analysis indicated that no studies were found to be removed since the estimate of each study when removed is within the confidence interval of the pooled analysis

The result of sensitivity analysis indicated that no studies were found to be removed since the estimate of each study when removed is within the confidence interval of the pooled analysis (Fig. 6).

### Factors associated with male involvement in PMTCT

The association between urban residence, partner knowledge in PMTCT, knowledge of ANC, partners have no negative perception towards for PMTCT, government employee, partners which were informed of need to go for PMTCT, health institution related barriers, primary education, and secondary education with male involvement in PMTCT were carried out. (Table.1).

**Table 1:** Factors associated with importance of male partner involvement in prevention of mother to child transmission of HIV/AIDS in Ethiopia: A Systematic review and Meta analysis from start of indexing until June, 2021

Variable name	No of study included	OR(95%CI)	Overall(I^2^,*P*-value)
Urban residence	2	2.433 (1.416–4.18)	74.1%, *p* = 0.239
partner knowledge of PMTCT	6	2.838 (1.907–4.224)	69.3%, *p* = 0.1641
Partner knowledge of ANC	3	3.495 (1.805–6.764)	76.8%, *p* = 0.014
Partners have no negative perception towards PMTCT	5	3.208 (2.180–4.721)	36.3%, *p *= 0.179
government employee	3	2.566 (1.756–3.750)	29.4%, *p* = 0.242
partners which were informed of the need to go for PMTCT	4	3.826 (1.879–7.792)	37.8% *p* = 0.200
Health worker-friendly approach	3	2.611(1.882–3.622)	0.0%, *p *= 0.724
Primary education	2	2.215 (1.289–3.807)	0.0%,*p* = 0.603
Secondary education	2	2.665 (1.692–4.196)	0.0%, *p* = 0.495

*ANC* Antenatal care, *CI* Confidence interval, OR Odds ratio, PMTCT Prevention of mother to child transmission

Urban residents were 2.4 times more involved in PMTCT than rural residents. Partners who had knowledge of PMTCT were 2.8 times more involved in PMTCT than poor knowledge. The odds of male involvement were 3.5 times higher among partners who had knowledge of ANC than no information. The absence of negative perception towards PMTCT increases male involvement in PMTCT by 3.2 times. Government employees were 2.5 times more involved in PMTCT than other workers. An informed partner who needs to go for PMTCT service was 3.8 times more involved than their counterparts. The smooth approach of health care providers increases male involvement in PMTCT by 2.6 times. Partners who had complete Primary and secondary education increase male involvement in PMTCT by 2.2 and 2.6 times respectively.

## Discussion

In the absence of any intervention, the incidence of HIV infection via mother-to-child transmission is estimated to be from 25 to 40% in developing countries [6]. In Ethiopia majority of pregnant women did not decide independently for acceptance of HIV testing. Decision-making authority is commonly referred to male partners [17]. This systematic review and Meta analysis were attempted to estimate the pooled prevalence of male involvement in PMTCT and factors associated with it.

The overall pooled prevalence of male involvement in PMTCT in Ethiopia is 31.82%; which is lower than WHO expectation [10] and finding in sub-Saharan Africa where male participation levels in hospital-based setting fall between 12.5% and 18.7% [18, 19]. This could be due to heterogeneity in the definition of independent and dependent variables and differences in a study setting; this review includes both community-based and hospital-based studies.

The odds of male involvement in PMTCT were 2.4 times higher among urban dwellers than rural residents. In rural settings poor roads, an underdeveloped transport system, and poor telecommunications typify the pervasive poverty [20]. Partners of rural residents are prone to the societal norm that men should not participate in PMTCT and believe that it is ridicule of men participating in ANC/PMTCT [21].

Partners who have knowledge of PMTCT were almost three times more involved in PMTCT. If partners have insufficient knowledge on PMTCT he fails to recognize the importance of male involvement for the Prevention of HIV infection from mothers to child or may have less access to sexual and reproductive health information [12]. The lack of knowledge about HIV and the importance of male involvement in PMTCT have direct implications for information, education, and communication initiatives. It highlights the need to increase male education on HIV/PMTCT and target information for men by various means [22, 23].

Partner's good knowledge of ANC services was about four times more likely to participate in PMTCT service than poor knowledge of ANC. Information on ANC could help women to take part in sexual and reproductive activity freely, encourage women to adopt HIV prevention practices, and helps to take action in transmission [24]. When the partner has information on ANC Counseling messages within ANC/PMTCT services could be easily for spousal communication regarding sexual risks [25]. It could also encourage women to discuss voluntary counseling and testing (VCT) of their spouses [26], and help them to elaborate plans to involve their partners early in PMTCT care service [27].

Partners who were informed of the need to have PMTCT were about four times more likely to involve in PMTCT than those not informed to have PMTCT. If the couples discuss the need for PMTCT partner were more likely to engage in adherence to the PMTCT treatment, modify infant feeding practice and increase condom use [10, 28].

Regarding the association of educational level and male involvement in PMTCT, a primary and a secondary level of education increases male involvement by 2.2 and 2.6 times respectively. In fact when the level of education increase individual understanding on mode of transmission and prevention strategy could increase [29, 30]. They might also have higher receptivity to new health-related information.

Partner perception towards PMTCT was another important variable identified in this review. Partners who have no negative perception towards PMTCT were almost three times more involved in PMTCT than their counterparts. There is a common social custom in many African countries that; ANC is women's affairs so men could not accompany their partners for maternal health care services [29]. Studies conducted in Uganda showed that cultural influence had an association with male involvement in PMTCT [31]. Systematic review conducted in sub-Saharan Africa revealed that PMTCT care and support are women’s work traditionally [19]. Similarly in Tanzania social and religious norm prohibit male involvement in female health services and the same is true in Cameroon, ANC activity was perceived by partners as outside their responsibility [20. 22].

Service delivery related factor helps to improve partner involvement in PMTCT. Most often resources are usually in short supply and antenatal clinics especially in developing countries are usually inadequately staffed [32]. This again decreases quality health care services since loaded care providers are less motivated. In this review when the attractive approach of providers increases male involvement by more than two times. However, there is contradicting finding in Nigeria that service-related factors have has no contribution to male involvement in PMTCT services [33]. Within the health system capacity reinforcement and motivation of the health service providers could improve the quality of services and minimize long waiting times within antenatal care [34].

This review reviled that partners whose occupation was government employee were about two times more likely to involve in PMTCT. Government employers were more likely to involve in PMTCT than a private employers [9].

This study has the following limitations, the actual number of men involvement was underreported since the review included studies done in women having health facilities visit and the review does not include qualitative data. The protocol for this systematic review and meta-analysis was not registered in the PROSPERO. The review provides useful information for policy and decision makers, local planners and health workers to give due attention to modify those multifaceted important factors affecting male involvement in PMTCT.

## Conclusions

The pooled estimate demonstrates that male involvement in PMTCT was low. This is far less than a 50% perception by the world health organization. Many predictors are affecting male involvement in PMTCT services. Minimizing these barriers in a step-wise manner would help to optimize the involvement of the male partner in PMTCT. Amendment of health care policy and context-specific adaptation help to achieve public health benefits. Stakeholders involved in this program should keep in mind that any effort to improve engagement of males in the Prevention of mother-to-child transmission would at the same time ensure that the reproductive rights of women are protected in full.

## Supplementary information

**Additional file 1. **Preferred reporting items for systematic review and Meta-Analysis items, 2020 guidelines with checklist.

**Additional file 2. **Examples of searches for PubMed and google scholar databases to assess the importance of male partner’s involvement in prevention of mother to child transmission of HIV/AIDS in Ethiopia: A systematic review and Meta analysis until June 2021.

**Additional file 3. Table S1.** JBI quality scores used to assess included article for importance of male partner’s involvement in prevention of mother to child transmission of HIV/AIDS in Ethiopia: A systematic review and Meta analysis until June 2021.

## Data Availability

The data sets generated during the current study are available from the corresponding author on request.

## Abbreviations

AOR: Adjusted odds ratio
AIDS: Acquired Immune Deficiency Syndrome
ANC: Antenatal Care
CI: Confidence Interval
HIV: Human Immune Deficiency Virus
PMTCT: Prevention of Mother to Child Transmission
PRISMA: Preferred Reporting Items for Systematic Reviews and Meta-Analyses
SNNPR: Southern Nation Nationality and Peoples Region
VCT: Voluntary Counseling and Testing
WHO: World Health Organization
