# Supplementary material for: Importance of male partner’s involvement in prevention of mother to child transmission of HIV/AIDS in Ethiopia: a systematic review and meta analysis until June 2021
Source: Arch Public Health. 2022 Oct 19;80:223. doi: 10.1186/s13690-022-00971-7 (PMC9580163; doi:10.1186/s13690-022-00971-7)
Supplement: Supplementary file 1 — Additional file 1. Preferred reporting items for systematic review and Meta-Analysis items, 2020 guidelines with checklist. [file 13690_2022_971_MOESM1_ESM.docx]

**
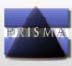
Additional file 1:** Preferred Reporting Items for Systematic Review and Meta- Analysis

items, 2020 guidelines with checklist.

| **Section and Topic** | **Item #** | **Checklist item** | **Line no** | **Page no** |
| --- | --- | --- | --- | --- |
| **TITLE** | | |  |  |
| Title | 1 | Identify the report as a systematic review. | Line 1-2 | 1 |
| **ABSTRACT** | | |  |  |
| Abstract | 2 | See the PRISMA 2020 for Abstracts checklist. | Line23-50 | 2-3 |
| **INTRODUCTION** | | |  |  |
| Rationale | 3 | Describe the rationale for the review in the context of existing knowledge. | Line 64-90 | 4-5 |
| Objectives | 4 | Provide an explicit statement of the objective(s) or question(s) the review addresses. | Line 99-105 | 5 |
| **METHODS** | | |  |  |
| Eligibility criteria | 5 | Specify the inclusion and exclusion criteria for the review and how studies were grouped for the syntheses. | Line 90-96 | 5 |
| Information sources | 6 | Specify all databases, registers, websites, organisations, reference lists and other sources searched or consulted to identify studies. Specify the date when each source was last searched or consulted. | Line108-113 | 6 |
| Search strategy | 7 | Present the full search strategies for all databases, registers and websites, including any filters and limits used. | Line 108-113 | 6 |
| Selection process | 8 | Specify the methods used to decide whether a study met the inclusion criteria of the review, including how many reviewers screened each record and each report retrieved, whether they worked independently, and if applicable, details of automation tools used in the process. | Line 115-122 | 6 |
| Data collection process | 9 | Specify the methods used to collect data from reports, including how many reviewers collected data from each report, whether they worked independently, any processes for obtaining or confirming data from study investigators, and if applicable, details of automation tools used in the process. | line 124-128 | 6 |
| Data items | 10a | List and define all outcomes for which data were sought. Specify whether all results that were compatible with each outcome domain in each study were sought (e.g. for all measures, time points, analyses), and if not, the methods used to decide which results to collect. | Line 130-132 | 7 |
|  | 10b | List and define all other variables for which data were sought (e.g. participant and intervention characteristics, funding sources). Describe any assumptions made about any missing or unclear information. | Line 130-132 | 7 |
| Study risk of bias assessment | 11 | Specify the methods used to assess risk of bias in the included studies, including details of the tool(s) used, how many reviewers assessed each study and whether they worked independently, and if applicable, details of automation tools used in the process. | Line 134-141 | 7 |
| Effect measures | 12 | Specify for each outcome the effect measure(s) (e.g. risk ratio, mean difference) used in the synthesis or presentation of results. | Line 149-150 | 7 |
| Synthesis methods | 13a | Describe the processes used to decide which studies were eligible for each synthesis (e.g. tabulating the study intervention characteristics and comparing against the planned groups for each synthesis (item #5)). | Line 99-105 | 5 |
|  | 13b | Describe any methods required to prepare the data for presentation or synthesis, such as handling of missing summary statistics, or data conversions. | Line 143 | 7 |
|  | 13c | Describe any methods used to tabulate or visually display results of individual studies and syntheses. | line143-144 | 7 |
|  | 13d | Describe any methods used to synthesize results and provide a rationale for the choice(s). If meta-analysis was performed, describe the model(s), method(s) to identify the presence and extent of statistical heterogeneity, and software package(s) used. | line 145-147 | 7 |
|  | 13e | Describe any methods used to explore possible causes of heterogeneity among study results (e.g. subgroup analysis, meta-regression). | line150-151 | 7-8 |
|  | 13f | Describe any sensitivity analyses conducted to assess robustness of the synthesized results. | Line 152 | 8 |
| Reporting bias assessment | 14 | Describe any methods used to assess risk of bias due to missing results in a synthesis (arising from reporting biases). | Line 153 | 8 |
| Certainty assessment | 15 | Describe any methods used to assess certainty (or confidence) in the body of evidence for an outcome. | line149-152 | 7-8 |
| **RESULTS** | | |  |  |
| Study selection | 16a | Describe the results of the search and selection process, from the number of records identified in the search to the number of studies included in the review, ideally using a flow diagram. | Line 157-163 | 8 |
|  | 16b | Cite studies that might appear to meet the inclusion criteria, but which were excluded, and explain why they were excluded. | fig 1 |  |
| Study characteristics | 17 | Cite each included study and present its characteristics. | Line165-171 | 8 |
| Risk of bias in studies | 18 | Present assessments of risk of bias for each included study. | 173-177 (Additional file 3) | 9 |
| Results of individual studies | 19 | For all outcomes, present, for each study: (a) summary statistics for each group (where appropriate) and (b) an effect estimate and its precision (e.g. confidence/credible interval), ideally using structured tables or plots. | Table 1 | 11 |
| Results of syntheses | 20a | For each synthesis, briefly summarise the characteristics and risk of bias among contributing studies. | Line 179-181 | 9 |
|  | 20b | Present results of all statistical syntheses conducted. If meta-analysis was done, present for each the summary estimate and its precision (e.g. confidence/credible interval) and measures of statistical heterogeneity. If comparing groups, describe the direction of the effect. | Line 183-185 | 9 |
|  | 20c | Present results of all investigations of possible causes of heterogeneity among study results. | Line 187-191 | 9 |
|  | 20d | Present results of all sensitivity analyses conducted to assess the robustness of the synthesized results. | Line 193-194 | 10 |
| Reporting biases | 21 | Present assessments of risk of bias due to missing results (arising from reporting biases) for each synthesis assessed. | 179-181 | 9 |
| Certainty of evidence | 22 | Present assessments of certainty (or confidence) in the body of evidence for each outcome assessed. | Line 197-209 | 10 |
| **DISCUSSION** | | |  |  |
| Discussion | 23a | Provide a general interpretation of the results in the context of other evidence. | Line 225-284 | 12-14 |
|  | 23b | Discuss any limitations of the evidence included in the review. | Line 285-287 | 14 |
|  | 23c | Discuss any limitations of the review processes used. | Line 287 | 14 |
|  | 23d | Discuss implications of the results for practice, policy, and future research. | line 291-298 | 15 |
| **OTHER INFORMATION** | | |  |  |
| Registration and protocol | 24a | Provide registration information for the review, including register name and registration number, or state that the review was not registered. | Line 299 | 15 |
|  | 24b | Indicate where the review protocol can be accessed, or state that a protocol was not prepared. |  |  |
|  | 24c | Describe and explain any amendments to information provided at registration or in the protocol. |  |  |
| Support | 25 | Describe sources of financial or non-financial support for the review, and the role of the funders or sponsors in the review. | Line 316 | 16 |
| Competing interests | 26 | Declare any competing interests of review authors. | Line 314 | 16 |
| Availability of data, code and other materials | 27 | Report which of the following are publicly available and where they can be found: template data collection forms; data extracted from included studies; data used for all analyses; analytic code; any other materials used in the review. | Line 311-312 | 16 |

**Additional file** 2: Examples of searches for PubMed and Google Scholar databases to assess the Importance of Male Partner’s Involvement in Prevention of Mother to Child Transmission of HIV/AIDS in Ethiopia: A Systematic review and Meta analysis until June 2021.

| Databases | Searching terms | Number of studies |
| --- | --- | --- |
| PubMed | (“*Partner” [All Fields]OR “partner”[MeSH terms]OR ”spouse”[all Fields] OR ”husband”[MeSH Terms]OR “husband”[All Fields]) AND (“involvement” [All Fields]OR “involvement”[MeSH terms]OR “participation”[All Fields]OR “include”[All Fields]) AND (“PMTCT”[All Fields]) AND( “associated”[All Fields] OR “determinants”[All Fields] OR “determinants”[MeSH terms])AND (“factors”[All Fields]OR “predictors”[MeSH terms]) AND ("ethiopia"[MeSH Terms] OR "ethiopia"[All Fields])* | 300 |
| Google scholar | “Prevalence” or “magnitude” or "incidence" and “associated factors” or "factors associated" or determinants" and “male involvement in PMTCT ” or "male participation” and” PMTCT” and “Ethiopia” | 25 |
| Other databases and Grey literatures | **Keywords searching**  Men’s involvement  Importance of Male partner involvements  Male and Prevention of mother to child transmission  Ethiopia | 5 |
| Total searched articles |  | 330 |
| Finally fulfill the eligibility criteria for our review |  | 10 |

**Additional file 3:** Table S1 JBI quality scores used to assess included article for Importance of Male Partner’s Involvement in Prevention of Mother to Child Transmission of HIV/AIDS in Ethiopia: A Systematic review and Meta analysis until June 2021.

| **Study ID** | **Author (year)** | **Weaknesses** | **JBI score** | **Quality status** |
| --- | --- | --- | --- | --- |
| 1 | Tilahun & Mohamed(2015) | No causal relationship of male involvement was established with prevention of mother to child transmission of HV. | 85% | Low risk |
| 2 | Lemma & Husein(2017) | The study considered small sample size.  No causal relationship of male involvement was established with prevention of mother to child transmission.  Risk factors were not exhaustively included | 58.5% | Low risk |
| 3 | Dagnew et al., (2020) | since it is community based study verbal autopsy was based on partners response whether he participate in PMTCT or not; so it is not like institution based study where possible to check when partners come up with his spouses or not | 64.5% | Low risk |
| 4 | Belato at al., (2016) | Factors were not exhaustively addressed and adjusted for confounding variables including male involvement | 60.5% | Low risk |
| 5 | Ayalew at al., (2020) | Relatively small sample size (420) even if the study conducted on two hospital( one comprehensive specialized hospital and one district hospital). | 75% | Low risk |
| 6 | Amsalu at a.,l (2013) | No causal relationship of male involvement was established with prevention of mother to child transmission of HIV.  Confidence interval for some variables remained wide. It might reflect the sample size (274) was not adequate as the study was community based longitudinal study. | 60.6% | Low risk |
| 7 | Amino & Musa (2014) | No causal relationship of male involvement was established with prevention of mother to child transmission of HV. | 87.5% | Low risk |
| 8 | Adane at al., (2020) | No causal relationship of male involvement was established with prevention of mother to child transmission of HV. | 84.6% | Low risk |
| 9 | Abuhay at al., (2014) | since it is community based study verbal autopsy was based on partners response whether he participate in PMTCT or not so it is not like institution based study where possible to observe he come up with his spouses or not | 75% | Low risk |
| 10 | Haile & Berhan (2014) | Factors were not exhaustively addressed and adjusted for confounding variables including male involvement | 70.5% | Low risk |
