# Supplementary material for: Importance of male partner’s involvement in prevention of mother to child transmission of HIV/AIDS in Ethiopia: a systematic review and meta analysis until June 2021
Source: Arch Public Health. 2022 Oct 19;80:223. doi: 10.1186/s13690-022-00971-7 (PMC9580163; doi:10.1186/s13690-022-00971-7)
Supplement: Supplementary file 3 — Additional file 3. Table S1. JBI quality scores used to assess included article for importance of male partner’s involvement in prevention of mother to child transmission of HIV/AIDS in Ethiopia: A systematic review and Meta analysis until June 2021. [file 13690_2022_971_MOESM3_ESM.docx]

Importance of Male Partner’s Involvement in Prevention of Mother to Child Transmission of HIV/AIDS in Ethiopia: A Systematic review and Meta analysis until June 2021

Shimeles Biru Zewude(SB) ^1*^ Asrat Hailu Dagne(AH) ^1^ Tewodros Magegnet Ajebe (TM)^2^

^1^Department of Midwifery, College of medicine and Health Sciences, Debre Tabor

University, Debre Tabor, Ethiopia

# ^2^Department of Midwifery, College Of Medicine and Health Sciences, Wollo University, Dessie,

# Ethiopia

^1^*Corresponding Authors: - Shimeles Biru zewude

nathanshimeles@gmail.com

**SB**: [nathanshimeles@gmail.com](mailto:nathanshimeles@gmail.com)

**AH**:1221asrat@gmail.com

**TM**: Ajebeteddy@gmail.com

**ABSTRACT**

**Background:** To promote the prevention of mother-to-child transmission (PMTCT) of HIV/AIDS male partners play a significant role, especially in developing country were men’s are a decision maker in domestic activity. When men are involved in PMTCT it would boost the service utilization and uptake of ART drugs. Thus this systematic review and Meta analysis aims to assess importance of male partner involvement in prevention of mother to child transmission of HIV/AIDS in Ethiopia.

**Methods**: Studies were accessed through an electronic web-based search mechanism from PubMed, Advanced Google Scholar, WHO databases and journals (African Health Monitor, Pan African Journal of Public Health), using independent and combinations of key terms together with a reference list of included studies. Two reviewers independently screened and assessed the quality of studies based on pre-specified criteria. When a disagreement between the two reviewers happened; the third reviewer was invited and resolve it based on the stated objectives and inclusion criteria. Measures of effects were pooled and random effect meta analysis was conducted.

**Results:** Ten studies met the inclusion criteria. The pooled prevalence of male involvement in PMTCT was 31.8% (95% CI; 22.3-41.3 I^2^ = 98.4%, p = 0.00). being urban residence(AOR= 2.43 95%CI;1.42-4.18), partner knowledge (AOR=2.84 95%CI;1.90-4.22), knowledge on Antenatal care(AOR=3.5 95%CI;1.80-6.76), partners who had no negative perception towards for PMTCT (AOR=3.21 95%CI;2.18-4.72), government employee(AOR=2.57 95%CI;1.76-3.75), partners informed of need to go for PMTCT(AOR=3.83 95%CI;1.88-7.79), health institution related barriers(AOR=2.6 95%CI;1.882-3.622), primary (AOR=2.21 95%CI;1.29-3.80), and secondary education(AOR=2.67 95%CI;1.69-4.19) were significant factors related with male partner involvement in prevention of mother to child transmission of HIV/AIDS.

**Conclusion:** the proportion of male involvement in the Prevention of mother-to-child transmission of HIV in Ethiopia was low. Interventions aimed at improving male participation in the Prevention of mother-to-child transmission should consider the factors related to it. Healthcare services may need to be inclusive and could help men active engagement in PMTCT programs.

**Keywords**: Male partner, Prevention of mother-to-child transmission of HIV, Meta analysis, Ethiopia

**BACKGROUND**

HIV/AIDS is one of the pandemic public health problems affecting many people including children. More than 38 million people globally were living with HIV in 2019[1, 2]. Worldwide 120,000 children died due to AIDS-related illnesses in 2016. This equates to 328 deaths every day. This is despite a 62% reduction in AIDS related death since 20 years ago. In the same year, 24% of pregnant women living with HIV did not have access to ARVs to prevent transmission to their infants and 160,000 children become infected with HIV [3]. Form the overall 90% of new infection believed to stem from mother to child transmission (MTCT) during pregnancy, childbirth and/or post-partum [4]

Male involvement has been recognized as a priority focus area to be strengthened in PMTCT but testing male partners for HIV in the context of preventing mothers to child transmission remains a challenge in most low and middle-income countries[5]. Male involvement has a great role in preventing women’s risk of infection by HIV, utilization on her PMTCT, to revive medication and helping women’s for proper infant feeding practices [6-7]. Another advantage of partner involvement in PMTCT is that there is an over-growing discordant rate among the couple. In Ethiopia, 5.6% of HIV negative urban dowelling married men are living with HIV infected partners and 2.2% of married HIV negative women are living with infected husbands, this implies that only screening the mother to prevent mother to child transmission of HIV doesn’t safeguard the child from acquiring HIV [8-9].

Countries are plan to nullifying mother to child transmission of HIV by 2030 [10]. To achieve the aforementioned goal male involvement is highly demanded. Poor male participation could also contribute to the problem as women in developing counties may depend on partners' decisions and support for both HIV testing and adherence to recommended PMTCT interventions [11]. Sub-optimal male participation is considered as a bottleneck for PMTCT programmatic achievement and has a negative bearing on women’s participation in voluntary HIV counseling and testing (HCT) and further program enrolment. Joint counseling and HIV testing for couples seem to be key to success in this regard [12]. At the national level there is no clear evidence that could be attributable factors associated with male partner involvement in the uptake of PMTCT services. Therefore, this systematic review and meta-analysis was aimed to address the following review question;

What is the proportion of male involvement in prevention of mother to child transmission of HIV/AIDS?

What are the factors associated with male partner involvement in the prevention of mother to child transmission of HIV/AIDS?

**METHODS**

**Inclusion and exclusion criteria**

The report was written by using preferred reporting items for systematic reviews and Meta analysis (PRISMA) guideline [13].(**additional file 1)** we considered articles published in the English language, have relevant full-text form, and studied in Ethiopia. There was no restriction on the publication period. The outcome was the proportion of male involvement in PMTCT as a decision-making tool, and its predictive factors, both published and unpublished studies at any time were included. However, studies available only as abstract with unclear outcomes, commentaries, editorials, and reviews were excluded.

**Information sources and search strategies**

Studies were accessed through the electronic web-based search mechanism from PubMed, Advanced Google Scholar, WHO databases and journals (African Health Monitor, Pan African Journal of Public Health). In addition, further search was made through snowballing or retrieving from relevant references used in related studies. A combination of medical subject headings (MeSH terms) using Boolean operators and keywords related to importance of Male partner involvement in PMTCT were used to search studies. We included articles published from inception until 10 June 2021.(**Additional file 2)**

**Study selection process**

Retrieved studies were exported to reference manager software, endnote version 6. Duplicated studies were removed using the endnote and manually. Two independent reviewers (SB) and (AH) screened the title and abstract for relevance. During this preliminary assessment, primary studies found to be irrelevant were excluded. When a disagreement between two reviewers happened, the third reviewer (TM) was used to handle the disagreement based on the relevance of the stated objectives and inclusion criteria. Finally studies with relevant information and fulfill inclusion criteria were selected for full-text review and excluded study were reason out via flow chart.

**Data collection process**

The data extraction form was prepared using an excel spreadsheet. Two independent reviewers extracted data by using a structured data extraction form. The name of the first author and year, region, study area, study design, sample size, determinants of male involvement, AOR (95% CI) were extracted. The extracted data then edited and saved in a comma-delimited file to suit for the analysis.

**Measurement of outcomes**

It has two main outcomes; proportion of male involvement in prevention of mother to child transmission of HIV/AIDS and the second one is importance of male partner involvement in prevention of mother to child transmission of HIV/AIDS. The odds ratio was calculated for the common risk factors of the reported studies.

**Study risk of bias assessment**

Two independent reviewers (SB) and (AH) appraised the quality of each study. The Joanna Briggs Institute (JBI) quality appraisal checklist was applied [14]. Data extraction was considered when studies scored above 50% for JBI quality indicators (**Additional file 3).** The inter-rater agreement between authors for study inclusion, data extraction, and methodological quality was assessed using Cohen’s kappa coefficient (values ≤ 0 as indicating no agreement and 0.01–0.20 as none to slight, 0.21–0.40 as fair, 0.41– 0.60 as moderate, 0.61–0.80 as substantial, and 0.81–1.00 as almost perfect agreement) [15]. Substantial agreement between reviewers, i.e., Cohen’s kappa coefficient > 0.72, was accepted.

**Data synthesis and analysis**

The data extracted and saved in comma-delimited file format in an excel spreadsheet was imported to Stata version 14.0 (Stata Corporation, College Station, TX, USA) statistical software for analysis. Heterogeneity among studies was checked using visual inspection of the results and using the I-squared (I^2^) statistic, in which 25, 50 and 75% represented low, moderate, and high heterogeneity respectively [16]. In this study I^2^ value of less than 50% was considered to interpret the combined effect size. Since there is heterogeneity between studies so Random effect model was used. Pooled analysis was done using mantel haenszel (M-H) statistical methods and effect measure was computed by odds ratio. Subgroup analysis was applied to investigate possible causes of statistical heterogeneity. Subgroup analysis was done by using publication year and sample size. Sensitivity analyses have been conducted to see the effects of a single study on determinants of male involvement in PMTCT. Potential publication bias was checked by using funnel plot and egger's regression test.

**RESULTS**

**Selection of studies**

A total of 330 studies were retrieved from sources by using pre-specified search strategies. All searched articles were exported to the endnote and then 80 were removed due to duplication. 250 Studies were screened for eligibility, relevance, accessibility, and outcome of interest. Accordingly, 220 studies were excluded due to inappropriate titles and were not examine factors or not focus on male involvement, and 15 is not original article and other reason. 5 articles were removed due to different outcomes of interest. Finally, 10 studies were included in the review. **(Fig.1)**

**Study characteristics**

Different factors such as urban residence, knowledge in PMTCT, partner knowledge of ANC, partners have no negative perception, government employee, partners which were informed of need to go for PMTCT, health institution related barriers, primary education, and secondary education were included in this study. A total of 4945 participants were included in this review. The sample size considered for primary studies ranges from 272 to 808. Regarding the study area, four of the studies were conducted at Amhara region, three in SNNPR (South Nation Nationalities and People region), and one in each region of Addis Ababa, Tigray, and Oromiya.

**Publication bias**

The funnel plot was assessed for asymmetry distribution of the magnitude of male involvement in PMTCT by visual inspection **(Fig.3)** and Egger’s regression test showed (P= 0.12) indicate no evidence of publication bias.

**Pooled estimate of male involvement in PMTCT**

The overall pooled prevalence of male partner involvement in PMTCT is presented with a forest plot **(Fig.2).** Therefore, the estimated proportion of men’s involved in PMTCT in Ethiopia was 31.82% (95% CI 22.31-41.33 I^2^=98.4%, P<0.000).

**Subgroup analysis**

Subgroup analysis was employed with the evidence of heterogeneity. In this study, the Cochrane I^2^ statistic was 98.4%, P < 0.000, which showed evidence of marked heterogeneity. Therefore, subgroup analysis was done using the publication year and sample size. As a result, male involvement in PMTCT is highest in the study conducted at or before 2015, 38.68%, whereas 32.16 % in sample size less than or equal to five hundred **(Fig. 4 and 5).**

**Sensitivity analysis**

The result of sensitivity analysis indicated that no studies were found to be removed since the estimate of each study when removed is within the confidence interval of the pooled analysis **(Fig.6).**

**Factors associated with male involvement in PMTCT**

The association between urban residence, partner knowledge in PMTCT, knowledge of ANC, partners have no negative perception towards for PMTCT, government employee, partners which were informed of need to go for PMTCT, health institution related barriers, primary education, and secondary education with male involvement in PMTCT were carried out. **(Table.1)**

Urban residents were 2.4 times more involved in PMTCT than rural residents. Partners who had knowledge of PMTCT were 2.8 times more involved in PMTCT than poor knowledge. The odds of male involvement were 3.5 times higher among partners who had knowledge of ANC than no information. The absence of negative perception towards PMTCT increases male involvement in PMTCT by 3.2 times. Government employees were 2.5 times more involved in PMTCT than other workers. An informed partner who needs to go for PMTCT service was 3.8 times more involved than their counterparts. The smooth approach of health care providers increases male involvement in PMTCT by 2.6 times. Partners who had complete Primary and secondary education increase male involvement in PMTCT by 2.2 and 2.6 times respectively.

Table 1: Factors associated with importance of male partner involvement in prevention of mother to child transmission of HIV/AIDS in Ethiopia: A Systematic review and Meta analysis from start of indexing until June, 2021:

| Variable name | No of study included | OR(95%CI) | Overall(I^2^,P-value) |
| --- | --- | --- | --- |
| Urban residence | 2 | 2.433 (1.416-4.18) | 74.1%, p = 0.239 |
| partner knowledge of PMTCT | 6 | 2.838 (1.907-4.224) | 69.3%, p = 0.1641 |
| Partner Knowledge of ANC | 3 | 3.495 (1.805-6.764) | 76.8%, p= 0.014 |
| partners have no negative perception towards PMTCT | 5 | 3.208 (2.180-4.721) | 36.3%, p=0.179 |
| government employee | 3 | 2.566 (1.756-3.750) | 29.4%, p = 0.242 |
| partners which were informed of the need to go for PMTCT | 4 | 3.826 (1.879-7.792) | 37.8% p=0.200 |
| health worker-friendly approach | 3 | 2.611(1.882-3.622) | 0.0%, p=0.724 |
| Primary education | 2 | 2.215 (1.289-3.807) | 0.0% ,p=0.603 |
| Secondary education | 2 | 2.665 (1.692-4.196) | 0.0%, p=0.495 |

ANC: Antenatal care CI: confidence interval OR: odds ratio PMTCT: prevention of mother to child transmission

**DISCUSSION**

In the absence of any intervention, the incidence of HIV infection via mother-to-child transmission is estimated to be from 25% to 40% in developing countries [6]. In Ethiopia majority of pregnant women did not decide independently for acceptance of HIV testing. Decision-making authority is commonly referred to male partners [17]. This systematic review and Meta analysis were attempted to estimate the pooled prevalence of male involvement in PMTCT and factors associated with it.

The overall pooled prevalence of male involvement in PMTCT in Ethiopia is 31.82%; which is lower than WHO expectation [10] and finding in sub-Saharan Africa where male participation levels in hospital-based setting fall between 12.5% and 18.7% [18-19]. This could be due to heterogeneity in the definition of independent and dependent variables and differences in a study setting; this review includes both community-based and hospital-based studies.

The odds of male involvement in PMTCT were 2.4 times higher among urban dwellers than rural residents. In rural settings poor roads, an underdeveloped transport system, and poor telecommunications typify the pervasive poverty [20]. Partners of rural residents are prone to the societal norm that men should not participate in PMTCT and believe that it is ridicule of men participating in ANC/PMTCT [21].

Partners who have knowledge of PMTCT were almost three times more involved in PMTCT. If partners have insufficient knowledge on PMTCT he fails to recognize the importance of male involvement for the Prevention of HIV infection from mothers to child or may have less access to sexual and reproductive health information [12]. The lack of knowledge about HIV and the importance of male involvement in PMTCT have direct implications for information, education, and communication initiatives. It highlights the need to increase male education on HIV/PMTCT and target information for men by various means [22-23].

Partner's good knowledge of ANC services was about four times more likely to participate in PMTCT service than poor knowledge of ANC. Information on ANC could help women to take part in sexual and reproductive activity freely, encourage women to adopt HIV prevention practices, and helps to take action in transmission [24]. When the partner has information on ANC Counseling messages within ANC/PMTCT services could be easily for spousal communication regarding sexual risks [25]. It could also encourage women to discuss voluntary counseling and testing (VCT) of their spouses [26], and help them to elaborate plans to involve their partners early in PMTCT care service [27].

Partners who were informed of the need to have PMTCT were about four times more likely to involve in PMTCT than those not informed to have PMTCT. If the couples discuss the need for PMTCT partner were more likely to engage in adherence to the PMTCT treatment, modify infant feeding practice and increase condom use [10, 28].

Regarding the association of educational level and male involvement in PMTCT, a primary and a secondary level of education increases male involvement by 2.2 and 2.6 times respectively. In fact when the level of education increase individual understanding on mode of transmission and prevention strategy could increase [29-30]. They might also have higher receptivity to new health-related information.

Partner perception towards PMTCT was another important variable identified in this review. Partners who have no negative perception towards PMTCT were almost three times more involved in PMTCT than their counterparts. There is a common social custom in many African countries that; ANC is women's affairs so men could not accompany their partners for maternal health care services [29]. Studies conducted in Uganda showed that cultural influence had an association with male involvement in PMTCT [31]. Systematic review conducted in sub-Saharan Africa revealed that PMTCT care and support are women’s work traditionally [19]. Similarly in Tanzania social and religious norm prohibit male involvement in female health services and the same is true in Cameroon, ANC activity was perceived by partners as outside their responsibility [20. 22].

Service delivery related factor helps to improve partner involvement in PMTCT. Most often resources are usually in short supply and antenatal clinics especially in developing countries are usually inadequately staffed [32]. This again decreases quality health care services since loaded care providers are less motivated. In this review when the attractive approach of providers increases male involvement by more than two times. However, there is contradicting finding in Nigeria that service-related factors have has no contribution to male involvement in PMTCT services [33]. Within the health system capacity reinforcement and motivation of the health service providers could improve the quality of services and minimize long waiting times within antenatal care [34].

This review reviled that partners whose occupation was government employee were about two times more likely to involve in PMTCT. Government employers were more likely to involve in PMTCT than a private employers [9].

This study has the following limitations, the actual number of men involvement was underreported since the review included studies done in women having health facilities visit and the review does not include qualitative data. The protocol for this systematic review and meta-analysis was not registered in the PROSPERO. The review provides useful information for policy and decision makers, local planners and health workers to give due attention to modify those multifaceted important factors affecting male involvement in PMTCT.

**CONCLUSIONS**

The pooled estimate demonstrates that male involvement in PMTCT was low. This is far less than a 50% perception by the world health organization. Many predictors are affecting male involvement in PMTCT services. Minimizing these barriers in a step-wise manner would help to optimize the involvement of the male partner in PMTCT. Amendment of health care policy and context-specific adaptation help to achieve public health benefits. Stakeholders involved in this program should keep in mind that any effort to improve engagement of males in the Prevention of mother-to-child transmission would at the same time ensure that the reproductive rights of women are protected in full.

# ABBREVIATIONS

AOR: adjusted odds ratio; AIDS: Acquired Immune Deficiency Syndrome; ANC: Antenatal Care; CI: Confidence Interval; HIV: Human Immune Deficiency Virus; PMTCT: Prevention of Mother to Child Transmission; PRISMA: Preferred Reporting Items for Systematic Reviews and Meta-Analyses; SNNPR: Southern Nation Nationality and Peoples Region; VCT: Voluntary Counseling and Testing; WHO: World Health Organization**.**

# DECLARATIONS

**Authors’ contributions**

SB: Conceived, designed, coordinated, searched, analyzed, interpreted data, and wrote the review and protocol. AH, and TM screened studies, extracted data, and appraised the quality of studies, and wrote the review. All authors read and approved the manuscript.

## Competing interests

All authors declare that they have no competing interests.

**Ethics approval and consent to participate**

Not applicable

**Consent for publication**

Not applicable

Availability of data and materials

The data sets generated during the current study are available from the corresponding author on request.

## Funding

No funding was obtained for this study.

**Acknowledgments**

We would like to thank all authors of the studies included in this systematic review and Meta-analysis.

**REFERENCES**

# 1. Joint Programme on HIV/AIDS and World Health Organization. AIDS epidemic update. Geneva, Switzerland: Joint UN Programme on HIV/AIDS and World Health Organization, 2007.

# 2. UNAIDS; 2020 fact sheet world AIDS day.

# 3.UNAIDS; 2017 ending AIDS progress towards the 90-90-90 target.

# 4. Family Health International. 2004. Preventing Mother-to-child transmission of HIV: A strategic framework. Institute for HIV/AIDS. Arlington, VA: FHI.

5. WHO, UNICEF (2007). New guidance on global scale-up of the prevention of mother to child

transmission of HIV: towards universal access for women, infants and young children and eliminating HIV/AIDS among Children. Switzerland: World Health Organization HIV/AIDS Department.

6. World Health Organization (2010).PMTCT Strategic Vision 2010– 2015. Preventing mother-

to child Transmission of HIV to reach the UNGASS and Millennium Development goals. Geneva, Switzerland: World Health Organization HIV/AIDS Department.

7. Peltzer K., Abbamonte JM., Mandell LN., Rodriguez VJ., Lee TK., Weiss SM., et al. The

effect of male involvement and prevention of mother-to child transmission (PMTCT)

Intervention on depressive symptoms in perinatal HIV-infected rural south African women. Archives of women's mental health in 2019.

8. HAPCO/ GAMET: HIV/AIDS in Ethiopia an epidemiological Synthesis, the Global

HIV/AIDS program. Addis Ababa Ethiopia; 2008. Available at http://site resources. World bank.org/INTHIVAIDS/Resources/375798-1103037153392/Ethiopia Synthesis final. pdf, Accessed in Janaury 2011.

9. Robert B., James T., Nulu S., Thorkild T: Determinants of male involvement in the prevention

of mother-to-child transmission of HIV programme in Eastern Uganda. BMC Reproductive Health 2010, 7(11). doi 10.1186/ 1742-4755-7-12.

10. World Health Organization (WHO): Male involvement in the prevention of mother-to-child

Transmission of HIV. 2012.

11. Antelman,G., Fawzi, MC., Kaaya, S., Mbwambo, J., Msamanga, GI., Hunter, DJ., et al.

Predictors of HIV-1 serostatus disclosure: a prospective study among HIV infected pregnant women in Dar es Salaam, Tanzania.2001 AIDS 15(14): 1865-74.

12. Theuring, S., Mbezi, P., Luvanda, H., Jordan-Harder, B., Kunz, A., et al. 2009. Male

Involvement in PMTCT services in Mbeya region, Tanzania. AIDS behavior 13 (suppl 1): S92-S102.

13. Preferred Reporting Items for Systematic Review and Meta-Analysis items (PRISMA 2020)

guidelines with checklist.

14. JBI. Joanna Briggs Institute reviewers’ manual: 2014 edition. Adelaide: JBI; 2014.

15. Landis, JR., Koch, GG. The measurement of observer agreement for categorical data.

Biometrics. 1977; 33(1):159–74.

16. Ioannidis, J., Patsopoulos, N., Evangelou, E. Uncertainty in heterogeneity estimates in meta-

analysis. BMJ. 2007;335:914–918.

17. Yohannes A (2014) Male involvement in prevention of mother to child transmission of HIV

and associated factors among males in Addis Ababa, Ethiopia. Am J Health Research 2: 338-343

18. RJ Akarro, M., Deonisia, FJ., Sichona. An evaluation of male involvement on the programme

for PMTCT of HIV/AIDS: a case study of Ilala municipality in Dar es salaam, Tanzania. Arts and Social Sciences Journal; 2011 ASSJ-201

19. John, D., Olivier, K., Richard M., Antoinette, T., Robert, C. Determinants of male

involvement in maternal and child health services in sub-Saharan Africans’ review.

Reproductive Health; 2012 ; 9:32.

20. Zegeye, E.A., Mbonigaba, J., & Dimbuene, Z.T. Factors associated with the utilization of

antenatal care and prevention of mother-to-child HIV transmission services in Ethiopia: applying a count regression model. BMC Women's Health **18,**187 (2018). <https://doi.org/10.1186/s12905-018-0679-9>

21. Morfaw et al.: Male involvement in prevention programs of mother to child transmission of

HIV: a systematic review to identify barriers and facilitators. Systematic Reviews 2013 2:5.

22. Nkuoh, GN., Meyer, DJ., Tih, PM., Nkfusai, J. Barriers to men’s participation in antenatal

And prevention of mother-to-child HIV transmission care in Cameroon, Africa. J Midwifery Womens Health 2010, 55(4):363–9.

23. Fisaha H. Male partner involvements in PMTCT. BMC Pregnancy and Childbirth

2014; 14:65.

24. Desgrees-Du-Lou, A, Brou, H., Djohan, G., Becket, R., Ekouevi, DK., et al: Beneficial

effects of offering prenatal HIV counseling and testing on developing an HIV preventive attitude among couples. Abidjan 2002– 2005. AIDS Behav 2009, 13:348–355. Ref Type: Generic

25. Farquhar, C., Kiarie, JN., Richardson, BA., Kabura, MN., John FN., Nduati, RW., et al:

Antenatal couple counseling increases uptake of interventions to prevent HIV-1 transmission. J Acquir Immune Defic Syndr 2004, 37(5):1620–6.

26. Msuya, SE., Mbizvo, EM., Hussain, A., Uriyo, J., Sam, NE., Stray-Pedersen, B: Low male

partner participation in antenatal HIV counseling and testing in northern Tanzania: implications for preventive programs. AIDS Care 2008, 20(6):700–9

27. Alemayehu, M., Etana, B., Fisseha, G., Haileslassie, K., Henock, Yebyo, YB. The role of

male partner involvement on mother’s adherence to PMTCT care and support, Tigray, Northern Ethiopia. Fam Med Med Sci Res. 2014;3(4):4–10. doi:10.4172/2327-4972.1000137

28. Sarker, M., Sanou, A., Snow, R., Ganame,J., and Gondos,A “Determinants of HIV

counselling and testing participation in a prevention of mother-to-child transmission programme in rural Burkina Faso,” TropicalMedicine and InternationalHealth, vol. 12, no. 12, pp. 1475–1483, 2007

29. Byamugisha,R., Tumwine, JK., Semiyaga, N., and Tyllesk¨ar,T. “Determinants of male

involvement in the prevention of mother-to-child transmission of HIV programme in Eastern Uganda: a cross-sectional survey,” Reproductive Health, vol. 7, no. 1, article 12, 2010.

30. Baiden,F., Remes, P., Baiden, R., et al., “Voluntary counseling and HIV testing for pregnant

women in the Kassena-Nankana district of northern Ghana: is couple counseling the way

forward?” AIDS Care, vol. 17, no. 5, pp. 648–657, 2005.

31. Godlove, N., Nkuoh, Dorothy, J., Meyer., Joseph, N., et al. Barriers to Men’s Participation

in Antenatal and Prevention of Mother-to-Child HIV Transmission Care, Cameroon, Africa;2010; 55:4.

32. E. Ademola, “Barriers to uptake of prevention of mother-to child transmission of HIV

services among mothers of vertically infected HIV-seropositive infants in,” Patient

Preference and Adherence, vol. 10, pp. 57–72, 2016.

33.Ditekemena, J., Matendo, R., Koole, O., Colebunders, R., Kashamuka, M., Tshefu, A., et al:

Male partner voluntary counselling and testing associated with the antenatal services in

Kinshasa, Democratic Republic of Congo: a randomized controlled trial. Int J STD AIDS 2011, 22(3):165–70.

34. Medley, A., Garcia-Moreno, C., McGill, S., Maman, S. Rates, barriers and outcomes of HIV

serostatus disclosure among women in developing countries: implications for prevention of mother-to-child transmission programmes. Bull World Health Organ 2004, 82(4):299–307.
